# A CRISPR/Cas9 Functional Screen Identifies Rare Tumor Suppressors

**DOI:** 10.1038/srep38968

**Published:** 2016-12-16

**Authors:** Alexandra Katigbak, Regina Cencic, Francis Robert, Patrick Sénécha, Claudio Scuoppo, Jerry Pelletier

**Affiliations:** 1 Department of Biochemistry, McIntyre Medical Sciences Building, McGill University, Montreal, Québec, H3G 1Y6, Canada; 2 Institute for Cancer Genetics, Columbia University Medical Center, 1130 St Nicholas Ave, New York, NY 10032, USA; 3 The Rosalind and Morris Goodman Cancer Research Center, 1160 Avenue des Pins, Montreal, Québec, H3A 1A3, Canada; 4 Dept of Oncology, 546 Pine Ave. West, Montreal, Québec, H2W 1S6, Canada

## Abstract

An enormous amount of tumor sequencing data has been generated through large scale sequencing efforts. The functional consequences of the majority of mutations identified by such projects remain an open, unexplored question. This problem is particularly complicated in the case of rare mutations where frequency of occurrence alone or prediction of functional consequences are insufficient to distinguish driver from passenger or bystander mutations. We combine genome editing technology with a powerful mouse cancer model to uncover previously unsuspected rare oncogenic mutations in Burkitt’s lymphoma. We identify two candidate tumor suppressors whose loss cooperate with MYC over-expression to accelerate lymphomagenesis. Our results highlight the utility of *in vivo* CRISPR/Cas9 screens combined with powerful mouse models to identify and validate rare oncogenic modifier events from tumor mutational data.

The International Cancer Genome Consortium is a colossal tumor sequencing endeavor that has profiled over 10,000 tumors and uncovered ~10 million mutations^1^. Mutation frequency, predicted functional impact, and pan-cancer analysis of mutated networks are powerful approaches by which to identify oncogenic drivers from this data in order to support diagnostic and therapeutic efforts^2^,^3^,^4^,^5^,^6^. However, cancers exhibit extensive mutational heterogeneity and in many cases it appears that only a few frequently mutated genes (among all tumor-associated mutations) are significant for initiation and progression. Indeed, the vast proportion of gene mutations within a tumor are thought to represent “passenger” or “bystander” mutations. However, it is unclear whether among these rarer events reside infrequent oncogenic drivers and this currently constitutes an obstacle to a full understanding of tumor biology.

Burkitt’s lymphoma (BL) is a common B-cell lymphoma, predominantly arising in children, which is characterized by the hallmark Burkitt translocation t(8;14)(q24;q32) or its variants t(2;8) and t(8:22) - all of which juxtapose the MYC oncogene with one of three immunoglobulin loci^7^. Recent whole genome, exome, and transcriptome sequencing data from 104 sporadic BL patient samples and BL cell lines has defined the mutational landscape in this cancer^8^,^9^,^10^. Among these studies, Schmitz *et al*.^8^ undertook RNA sequencing of 28 sporadic BL samples and 13 cell lines and identified >5000 mutations, Love *et al*.^9^ identified 70 recurrently mutated genes from exome sequencing of 51 primary BL tumors and 8 BL cell lines and Richter *et al*.^10^ sequenced four Burkitt’s lymphomas and identified 119 genes with potentially protein-altering mutations. Within this rich source of BL mutational data lie known oncogenic drivers alongside a large number of infrequently mutated genes, leading to a characteristic “long tail” phenomenon when analyzing gene mutation counts in tumors (see below). The significance of this latter class of mutations in BL remains unknown and it is here that functional assays have much to offer.

## Results

### Coupling CRISPR/Cas9 and the E*μ*-Myc model to identify rare modifiers of tumor formation in BL

**Figure 1:**
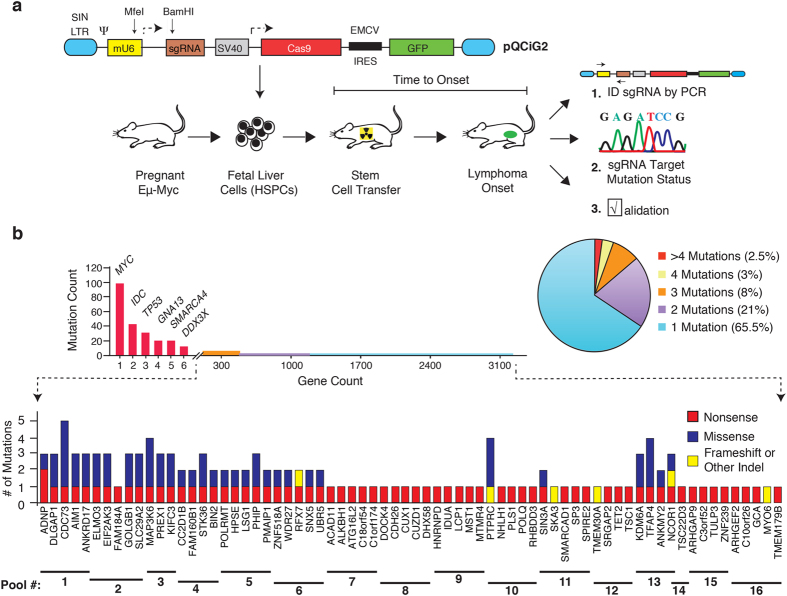
(**a**) Schematic representation of adoptive transfer strategy workflow used for Cas9/sgRNA delivery to HSPCs, followed by transplantation and lymphoma monitoring. Details of pQCiG2 have been previously reported^18^. Accelerated tumors were characterized by: (1) PCR amplification and sequencing of sgRNAs residing in the resulting tumors, (2) the sgRNA targeted loci were probed for mutational status in the obtained tumors, and (3) independent sgRNAs and shRNAs were used in new transplantation experiments for validation of results. (**b**) Gene mutation count in BL. The top graph represents all coding region mutations, as reported in Love *et al*.^9^ (Supplementary Table 3), Richter *et al*.^10^ (Supplementary Table 9) and Schmitz *et al*.^8^ (Supplementary Table 1). The right pie chart denotes the proportion of genes harboring the indicated number of mutations found in the 104 BLs. The bottom graph highlights the frequency and nature of mutations within genes in which at least one nonsense or frameshift mutation was identified in BL mutational studies. Gene names and sgRNA pools are indicated at the bottom. One sgRNA was omitted from pools 2–4, while 3 were omitted from pool 14 as a consequence of quality control experiments revealing that the original vector contained undesired second site mutations.

To functionally screen for rare modifiers of tumor formation from BL sequencing data, we took advantage of an adoptive transfer strategy utilizing the E*μ*-Myc genetically engineered mouse model (GEMM) (Fig. 1a). This GEMM is modeled after the defining Burkitt’s translocation and recapitulates typical genetic and pathological features of human non-Hodgkin’s lymphomas^11^,^12^. It has been extremely useful for unraveling oncogene cooperation, defining pathway addictions, and elucidating drug response/genotype relationships *in vivo* in cancer^13^. From the large number of rarely mutated genes in BL, we focused on genes that had incurred nonsense or frameshift mutations and thus could easily be disrupted using CRISPR/Cas9 (Fig. 1b, Supplementary Figure 1a, and Supplementary Table S1)^8^,^9^,^10^. Perusal of the human BL mutation data identified 91 genes fulfilling this criteria, although in many cases, additional missense mutations were noted in independent BL samples (Fig. 1b and Supplementary Table S1). Our screen focused on genes not known to be modifiers in this cancer type and that had not been previously characterized in BL. A few well characterized tumor suppressors were retained (e.g. *Tsc1, TP53*) and served as positive controls in our assay^14^,^15^. In total, 75 sgRNAs targeting the murine orthologs of genes infrequently mutated in BL were generated (Supplementary Table S1). We designed the sgRNAs to target their murine counterpart in the vicinity of the nonsense or indel mutation that had been documented in the human BL data. Testing of 9 randomly chosen sgRNAs indicated that all displayed significant editing activity, as assessed by the T7EI cleavage assay (Supplementary Figure 1b).

One of the parameters that we wished to define before undertaking an *in vivo* screen in the E*μ*-Myc model was to elucidate the sgRNA pool complexity that would enable identification of “hits” following reconstitution of hematopoietic stem and progenitor cells (HSPCs) in transplanted recipients (Fig. 1a). To this end, we used a well characterized p53-targeting sgRNA, sgp53-1 and an sgRNA targeting the neutral Rosa26 locus as positive and negative controls, respectively^16^,^17^. All sgRNAs were co-expressed with Cas9 from a second generation “All-in-One” retroviral vector that also produced green fluorescent protein (GFP), enabling tracking of infected cells by flow cytometry (Fig. 1a)^18^.

E*μ*-Myc HSPCs transduced with undiluted sgp53-1, or with a 1:5 dilution of sgp53-1 in sgRosa26, produced tumors in recipients by ~25 days with complete penetrance (Supplementary Figure 2a). Mice receiving HSPCs with sgp53-1 diluted 1:20 or 1:100 developed tumors with a slightly slower onset rate and with incomplete penetrance. In contrast, E*μ*-Myc HSPCs transduced with undiluted sgRosa26 produced tumors with a median onset rate of ~80 days (Supplementary Figure 2a). These results indicate that a functional sgRNA targeting a tumor suppressor gene could be reproducibly enriched from pools containing 5 different sgRNAs.

### *In vivo* screening identifies candidate sgRNAs capable of promoting lymphomagenesis

**Figure 2:**
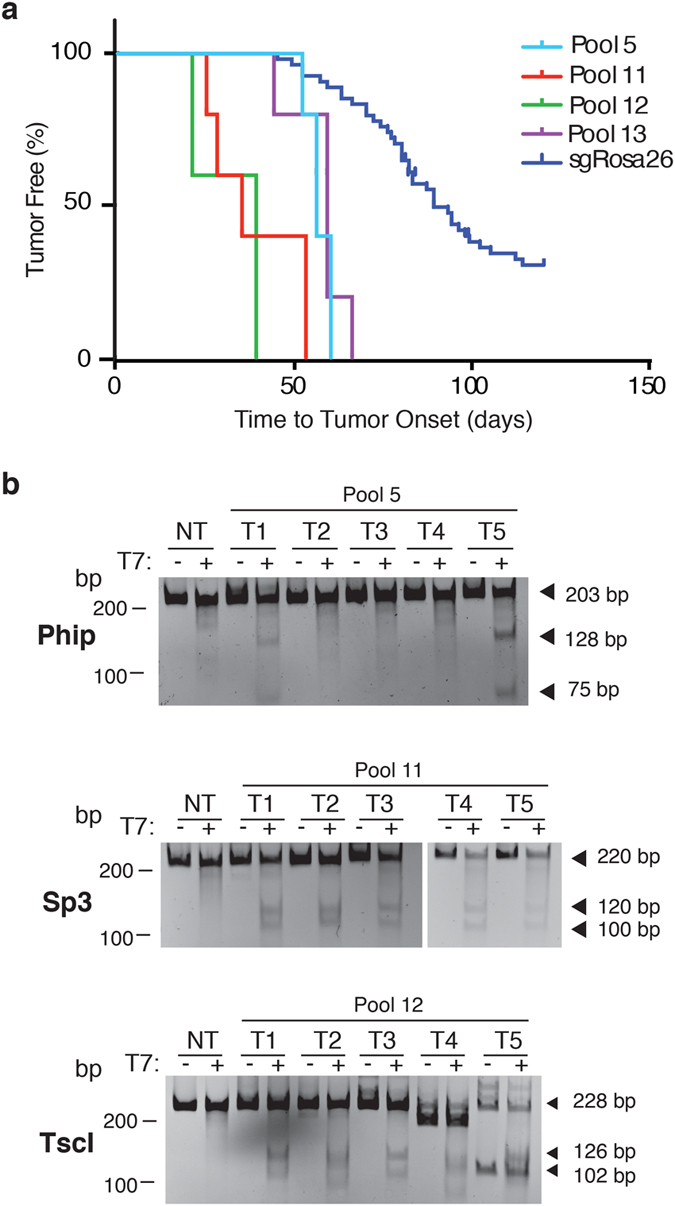
sgRNA pools exhibiting accelerated tumorigenesis. (**a**) Kaplan-Meier plot of tumor onset rates in mice transplanted with HSPCs infected with the indicated sgRNA pools. Note that data from all cohorts receiving sgRosa26 are combined and used as reference in these plots. (**b**) T7EI assay from individual tumors of the indicated pools or non-targeted (NT) control cells. The locus targeted for amplification is shown to the left of each gel.

Based on the results of our dilution experiments, we screened our candidate genes in pools maximally containing five sgRNAs (Fig. 1b and Supplementary Table S1). This yielded a total of 16 pools that were used to transduce at least three independent HSPC populations and transplanted into five irradiated recipients. Four of the pools showed significantly increased tumor onset rates compared to mice having received HSPCs infected with pQCiG2/sgRosa26 (Fig. 2a; p < 0.0001, (Log-Rank Mantel-Cox Test)). None of the recipients receiving HSPCs infected with the other pools developed lymphomas at rates that were significantly different than those obtained with pQCiG2/sgRosa26 (Supplementary Figure 2b).

Despite the presence of a GFP reporter within our transduction vector, we noted that not all recovered tumors were GFP^+^, which we attributed to the absence of selective pressure to maintain expression from pQCiG2 following locus modification. To identify the tumor-promoting sgRNAs in tumors arising from sgRNA pools 5, 11, 12, and 13, we isolated genomic DNA from all lymphomas, amplified the sgRNA encoding sequences by PCR, and sequenced the amplified products. Two of five tumors from Pool 5 yielded PCR products that, when sequenced, revealed the presence of sgRNAs targeting only *Phip* (data not shown). T7EI analysis of the *Phip* locus in tumors revealed the presence of mutations at the *Phip* locus in those same two tumors (Fig. 2b, Top panel: T1 and T5). We have not further characterized the three remaining tumors (T2, T3, T4) to determine the underlying oncogenic event since we failed to retrieve PCR products from these tumors and cannot exclude that these tumors arose independently of CRISPR-induced mutagenesis. From Pool 11, 5/5 tumors revealed the presence of only *Sp3*-targeting sgRNA and T7EI analysis of these 5 tumors confirmed modification at the endogenous *Sp3* locus (Fig. 2b, Middle panel). All tumors from Pool 12 harbored an sgRNA targeting *Tsc1* and indeed all 5 tumors showed evidence of mutagenesis at this locus (Fig. 2b, bottom panel). *Tsc1*, as well as *Tsc2*, has been previously shown to accelerate lymphomagenesis in the E*μ*-Myc model and was not further pursued^15^,^19^. All tumors from Pool 13 revealed the presence of an sgRNA targeting *Tfap4* (data not shown).

### *In vivo* validation of candidate tumor suppressors

**Figure 3:**
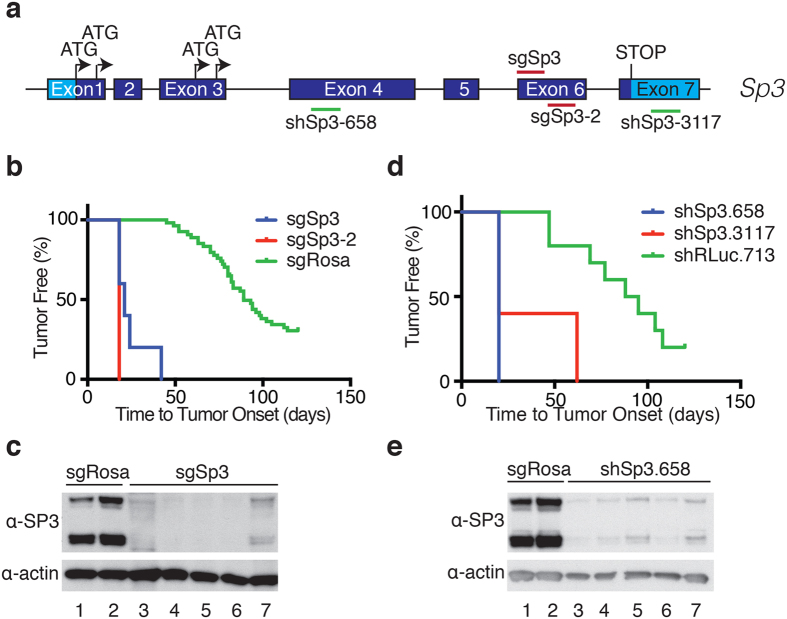
Validation of *Sp3* as a tumor suppressor. (**a**) Schematic of the *Sp3* gene regions targeted by the sgRNAs and shRNAs used in this study. Note that 4 protein isoforms are generated from the *Sp3* gene as a result of alternative translation initiation events^43^. The light blue shading represents untranslated regions whereas dark blue denotes coding exons. (**b**) Kaplan-Meier plot of tumor onset rates in mice receiving HSPCs infected with retroviruses expressing the indicated sgRNAs. Data from all cohorts receiving sgRosa26 are combined and used as reference in this plot. (**c**) Immunoblots comparing SP3 levels in tumors obtained from mice transplanted with HSPCs infected with Cas9/sgRosa26 or Cas9/sgSp3. (**d**) Kaplan-Meier plot of tumor onset rates in mice receiving HSPCs transduced with retroviruses expressing the shRNAs targeting RLuc (neutral control) or *Sp3*. Data from all cohorts receiving shRLuc.713 were pooled and used as reference. (**e**) Immunoblots assessing SP3 protein levels in sgRosa26- or shSp3.658-derived tumors.

**Figure 4:**
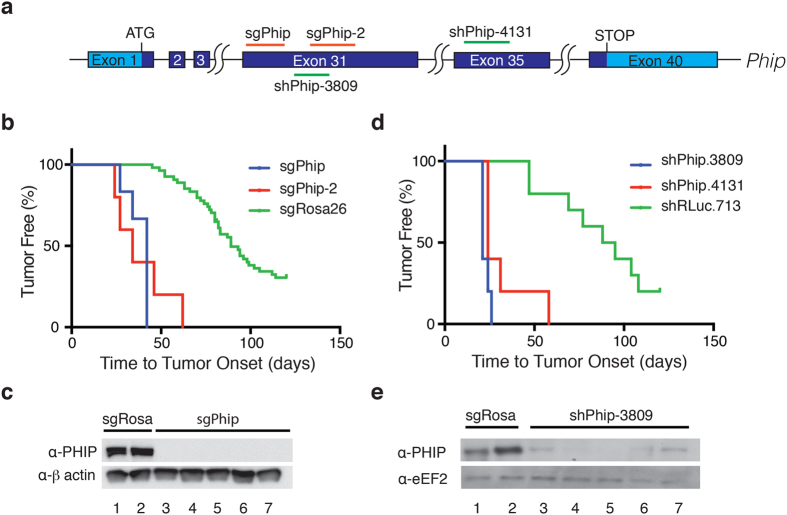
Validation of *Phip* as a tumor suppressor. (**a**) Schematic diagram of the *Phip* gene indicating the sgRNA and shRNA targeted sites. The light blue shading represents untranslated regions whereas dark blue denotes coding exons. (**b**) Kaplan-Meier plot of tumor onset rates in mice receiving HSPCs infected with retroviruses expressing the indicated sgRNAs. Data from all cohorts receiving sgRosa26 are combined and used as reference in this plot. (**c**) Immunoblots comparing PHIP levels in sgPhip and sgRosa26 derived tumors. (**d**) Kaplan-Meier plot of tumor onset rates in mice receiving HSPCs transduced with retroviruses expressing shRNAs targeting *Phip*. Data from all cohorts receiving shRLuc.713 were pooled and used as reference. (**e**) Immunoblots comparing PHIP levels in sgRosa26- or shPhip.3809-derived tumors.

We undertook to validate these results by repeating the HSPC adoptive transfer experiment using only the original sgRNA as well as a second independent, non-overlapping sgRNA (Figs 3 and 4 and Supplementary Table S2). For *Sp3* and *Phip*, both sgRNAs lead to increased lymphoma onset compared to the Rosa26 cohort (Figs 3b and 4b). For *Tfap4*, we were able to recapitulate accelerated tumorigenesis with the original sgRNA, but not with a second independent sgRNA (data not shown) and thus did not further pursue *Tfap4* characterization. Sequencing of cloned amplicons obtained from PCR amplification across the sgRNA targeted loci for *Sp3* and *Phip* from tumors obtained in the validation experiment revealed indel mutations (Supplementary Figures 3 and 4). We also noted considerable sequence heterogeneity at the *Sp3* or *Phip* loci within any given lymphoma indicating that the tumors that arose were polyclonal in nature and thus unlikely to be due to rare integration events that inactivated a tumor suppressor locus. Western blot analysis of tumors obtained generated by CRISPR/Cas9 targeting of *Sp3* and *Phip* indicated significant reductions in levels of both proteins in all tumors analyzed (Figs 3c and 4c). We attribute the small levels of residual protein to normal cells contaminating the tumor samples.

### SP3 and PHIP display tumor suppressive activity *in vivo*

Both SP3 and PHIP have been reported to exhibit pro-oncogenic activity in some contexts (see Discussion) and yet, in the E*μ*-Myc model we clearly identified these as tumor suppressors. Therefore, to strengthen our results and also exclude the possibility that modification of the *Sp3* or *Phip* loci by CRISPR/Cas9 had led to the generation of gain-of-function truncation mutants, we targeted *Sp3* and *Phip* for knockdown using two independently generated shRNAs. Our rationale was that if loss of function was truly responsible for tumor initiation in our Cas9-based experiments (Figs 3b and 4b), we should be able to phenocopy this using shRNAs to reduce gene activity. In both cases, using two independent shRNAs, we observed significantly accelerated tumor onset as compared to a neutral control shRNA targeting renilla luciferase (Figs 3d and 4d). As expected, the resulting tumors showed significant reductions in target protein expression levels (Figs 3e and 4e). As well, ectopic expression of a PHIP C-terminal truncation mutant, lacking the same functional regions as predicted from the human mutation identified in BL, did not lead to accelerated tumorigenesis following infection of E*μ*-Myc HSPCs (Supplementary Figure 5). In sum, our results demonstrate that both *Sp3* and *Phip* behave as tumor suppressors in E*μ*-Myc driven lymphomas.

## Discussion

Our results provide a framework for identifying functionally relevant rare mutations in human tumor sequencing data. Our approach is complementary to bioinformatics initiatives that score for mutation frequency and predicted gene function to identify modifiers of tumor formation among mutational data. Interestingly, perusal of the Catalogue of Somatic Mutations in Cancer (COSMIC) database revealed that *PHIP* and *SP3* are found mutated in other human cancers, including a small fraction of hematopoietic and lymphoid cancers (Supplementary Figure 6).

Both pro-survival and pro-apoptotic properties have been attributed to *Sp3*, making it impossible to predict its roles in tumor initiation simply based on perusal of the literature. Sp3 has been identified as a component of the transcriptional regulatory network activated by MYC overexpression^20^. As well, both MYC and SP3 are involved in upregulation of the telomerase catalytic subunit, TERT^21^ a process whose dysregulation is known to drive B-cell leukemia^22^. SP family members SP1/3/4 have been implicated as non-oncogenic addiction events in pancreatic cancer xenograft experiments^23^. Yet, reduced SP3 expression is associated with decreased apoptosis-related caspase activity^24^ and decreased oncogenicity^25^,^26^,^27^ whereas elevated levels in LS174 modified colon carcinoma cells leads to increased apoptosis and prevents tumor formation in nude mice^28^.

Similar conflicting data exists for *Phip.* Increased *Phip* copy number is correlated with ulceration in melanoma^29^ and increased PHIP expression is linked to increased likelihood of metastasis and poor prognosis in melanoma patients; whereas knockdown of *Phip* in mouse models prolongs survival and has been reported to protect against metastasis^30^. In the E*μ*-Myc model, PHIP and SP3 demonstrated clear *in vivo* tumor suppressor activity (Figs 3 and 4). This may reflect context-dependency of tumor-suppressor activity as exemplified by eIF5A, which acts as a promoter of oncogenesis in a liver cancer model^31^, but when knocked-down in the Eμ-Myc system, acts as a potent tumor suppressor^32^.

The possibility of context-dependent effects of PHIP and SP3 mutation is also supported by data summarized by the cBioPortal for cancer genomics (http://www.cbioportal.org/) where 27.6% of cases in a breast cancer xenograft model show increased PHIP copy number, while 14.6% of DLBCL cases reported by the Cancer Genome Atlas (TCGA) have deletions of PHIP^33^,^34^ Although less frequently reported as being altered in cancers than PHIP, differences in copy number variation of SP3 are also seen depending on the tissue examined, with a majority of alterations in prostate samples reported by TCGA as deletions, while ovarian and breast cancers more frequently display amplifications^33^,^34^. CRISPR/Cas9 in concert with available deep-sequencing data and an appropriate GEMM is thus a powerful approach to identify context-dependent lesions.

CRISPR/Cas9 is well-suited for the type of *in vivo* loss-of-function screens undertaken herein, but we note that our screen was not exhaustive. Expanding the number of sgRNAs used per gene, increasing the animal cohort size, and technological improvements should improve the discovery rate and throughput of the screen. Related to the latter point - an obvious limitation of this screen is the fairly low complexity of sgRNA screening pools used herein as compared to previously published shRNA screens^32^. One limitation is the large size of the pQCiG2 sgRNA/Cas9 retroviral delivery vector (~8 kbp) which leads to reduced viral titers and subsequent lower infection efficiencies^35^. Hence the incorporation of Cas9 alleles into cancer GEMMs^36^ will allow the use of smaller sgRNA delivery vectors with higher viral titers and hence, transduction efficiencies.

Although BL and the E*μ*-Myc model share the same initiating genetic lesion (i.e. a translocation leading to elevated MYC expression), the etiology of the murine and human diseases differ. As the translocation is present in the germline in the E*μ*-Myc model, transformation arises in pro- and pre-B cells in the bone marrow at a time when the E*μ* enhancer becomes activated and begins driving MYC expression. Human BL however arises as a consequence of a *Myc* translocation occurring in more mature B cells present in lymph node germinal centers. This may be a limitation of the E*μ*-Myc model and we may be under-estimating the number of oncogenic lesions in BL if any of these are B cell-stage specific. Nonetheless the E*μ*-Myc model has proven itself as an excellent genetic system for identifying lesions that co-operate with MYC *in vivo*^14^. This model has correctly reported on the ability of *p53* suppression^37^ or *Tsc1 and Tsc2* loss^15^ to accelerate tumorigenesis - genes mutated in human BL. Given that the Tsc1/2 complex regulates mTORC1 activity, and that mTORC1 is a drug target for treatment of B-cell lymphomas^38^, these results highlight the potential of our approach for identifying therapeutic targets.

In the era of personalized medicine, the identification of rare alleles that restrict tumorigenesis has important therapeutic implications. If some of the identified genes are pro-oncogenic in certain settings and tumor suppressive in others, defining context is critical to supporting correct clinical drug development. Also, understanding downstream networks perturbed by loss of PHIP or SP3 could lead to identification of new therapeutic targets. Rare mutational events may dilute the response to therapeutics targeting the more frequent mutational events and a better understanding of these events will enable better clinical stratification. If loss of PHIP or SP3 is also required for tumor maintenance, then this would support the rationale for biotherapeutic development, such as approaches aiming to systemically deliver wild-type protein^39^. The priority placed on developing tailored therapeutics to rare mutations that can drive tumorigenesis will ultimate be determined by their overall relevance to tumor biology.

## Methods

### Retroviral Infections, Stem Cell Isolation, and Adoptive Transfer

Low passage Phoenix-Eco viral packaging cells were cultured in complete DMEM (10% FBS, 1% Penicillin-Streptomycin, 1% L-Glutamine) at 37 °C in 5% CO_2_. Twenty-four hours prior to transfection, 3.5 × 10^6^ cells were seeded in 10 mL DMEM in 10 cm tissue culture plates. pQCiG2 constructs were pooled in equal molar ratios to a total of 10 *μ*g and co-transfected into Phoenix-Eco cells with 1 *μ*g pCL-eco replication-incompetent helper vector^40^ using calcium phosphate. Twenty-four hours after transfection and twelve hours before the first virus harvest infection, plates were washed with PBS and refreshed with 5 mL complete BCM (45% DMEM, 45% IMEM, 10% FBS, 1% Penicillin-Streptomycin, 1% L-Glutamine). Virus was collected 4 times, every 12 hrs starting from 12 hrs after BCM (B cell media) media change.

Hematopoietic stem and progenitor cells (HSPCs) were isolated from fetal livers at E13.5 and frozen until used. Cells were thawed 12 hrs before first infection in BCM supplemented with 1 ng/mL IL-3, 10 ng/mL IL-6, 100 ng/mL SCF (stem cell factor) and incubated at 37 °C in 5% CO_2_. Cultured HSPCs were infected four times at 12 h intervals with viral supernatant from transfected Phoenix-Eco cells, supplemented with 1 ng/mL IL-3, 10 ng/mL IL-6, 100 ng/mL SCF and 4 μg/mL Polybrene, and spinoculated at 950 × g for 1 h at 37 °C. Transduction efficiency was assessed by determining the GFP^+^ population by flow cytometry using a Guava 8HT flow cytometer (Millipore).

For transplantations, 6–8 week old female C57BL/6 mice were placed on 0.125 mg/mL ciprofloxacin + 2% sucrose two days before transplantation. Four hours before transplantation, mice were irradiated with 4 Gy of γ radiation. Approximately 6 × 10^5^–8.2 × 10^5^ cells were transplanted into irradiated mice via intravenous tail-vein injection. Mice were maintained on antibiotics for 3 weeks post-transplantation. Mice were palpated twice a week to assess tumor status until the experimental end point at day 120. When tumors arose, mice were sacrificed and the masses harvested. Lymphomas were gently macerated between the frosted ends of two microscope slides and the resulting cell suspension was passed through a 40 *μ*m cell strainer to isolate single cells. These cells were then frozen in BCM + 20% FBS + 10% DMSO and stored in liquid N_2_ until further used. All methods were performed in accordance with the relevant McGill guidelines and regulations, including licensing for use of biohazard material. In addition, all animal studies were approved by the McGill University Faculty of Medicine Animal Care Committee.

### Recovery of sgRNAs and T7 Endonuclease I Assay (T7EI)

Genomic DNA was prepared from isolated tumor cells by lysing tumor cell pellets overnight in TNE buffer (10 mM Tris [pH 8.0], 100 mM NaCl, 25 mM EDTA [pH 8.0], 0.25% SDS, 125 *μ*g/mL Proteinase K, 125 *μ*g/mL RNase A) at 55 °C. Genomic DNA was deproteinized by extracting once with phenol, twice with phenol:chloroform: (50:50), and once with chloroform and recovered by ethanol precipitation using 0.3 M NaOAc [pH 5.2]. PCR amplification of targeted loci was performed using Phusion High-Fidelity DNA polymerase (NEB) according to the manufacturer’s recommendations. Amplified DNA was purified using BioBasic EZ-10 spin columns. The T7EI assay was then performed as previously described^16^ and the entire reaction was resolved on a 15% 1× TBE polyacrylamide gel (29:1 acrylamide:bisacrylamide) before staining with ethidium bromide.

### Sequencing of Modified Loci

Targeted loci were amplified from tumor genomic DNA using primers designed with Primer3^41^ and containing adaptor sequences (Supplementary Table S3). The amplified loci were then cloned into pSKII(+) and inserts sequenced via Sanger sequencing using the T7 sequencing primer.

### Small Hairpin (sh) RNA Design

The Designer of siRNA (DSIR) algorithm with extended rules described by Fellman *et al*.^42^ was used to generate shRNAs targeting *Phip* and S*p3*. Five shRNAs targeting each gene were generated and cloned into the MLS retroviral backbone using unique *XhoI/EcoRI* restriction sites. After validation *ex vivo* in cell lines, the two most potent shRNAs were chosen for use in HSPC adoptive transfer experiments.

### PHIP and PHIP ^R1212Δ^ Plasmids

The PHIP cDNA was kindly provided by Dr. Anne-Claude Gingras (The Lunenfeld-Tanenbaum Research Institute, Toronto). From this cDNA, a truncation mutant was generated by excising the C-terminal region of PHIP using unique AgeI/XhoI sites and replacing it with an oligonucleotide containing a premature stop codon to generate PHIP ^R1212Δ^. For insertion into MLS, the proviral backbone was digested with *BglII*, repaired with Klenow, and digested with *XhoI*. PHIP and PHIP ^R1212Δ^ were excised from the parental plasmid by digestion with *AscI*, klenow repaired, and digested with *XhoI*. Following gel purification, the PHIP cDNAs were ligated into MLS and the integrity of the resulting clones verified by sequencing.

### Antibody Generation and Western Blotting

The DNA sequence encoding amino acids 661-913 of PHIP (Uniprot: Q8VDD9) was amplified from the complete cDNA with PCR Primers 5′GAATTCGAAGCAGGTGTTAGTAATGCCAG3′ and 5′CTCGAGTCACTTTGGTGATGTTGGTCCATC3′. This product was then cloned into pSKII(+) before subcloning into pGEX6P1 using unique EcoRI/XhoI restriction sites, which allowed the in-frame addition of a GST tag to the N-terminus of the protein. The GST-fusion protein was then purified from BL21 *E. coli* induced with 0.3 mM IPTG for 4 hours. Bacteria were lysed in 1 M NaCl, 50 mM Tris –HCl [pH 8.0], 1 mM EDTA [pH 8.0], 1 mM EDTA and protein was purified with Gluthatione Sepharose 4B (Amersham) before eluting with 50 mM Tris [pH 7.5], 10 mM reduced Glutathione. Proteins were dialyzed and stored in 50 mM Tris [pH 8.0], 150 mM NaCl, 10 mM EDTA,1 mM DTT, and 20% Glycerol. The GST tag was cleaved from the purified PHIP antigen using GST-3C protease followed by subsequent retrieval from the flow-through following passage through a Glutathione Sepharose column. The resulting protein was used as antigen for subsequent immunizations.

Protein extracts for immunoblotting were prepared by lysing tumor cell pellets in RIPA buffer (20 mM Tris-HCl [pH 7.5], 150 mM NaCl, 0.1% SDS, 1% NP40, 0.5% sodium deoxycholate, 1 mM β-glycerophosphate, 1 mM PMSF, 1 *μ*g/ml leupeptin, 10 *μ*g/ml aprotinin, and 2.5 *μ*M pepstatin A) on ice for 10 minutes, followed by sonication. Extracts were then boiled for 10 minutes at 95 °C in 1X Laemmli sample buffer and resolved on a 6% or 8% NuPAGE gel. Proteins were transferred to PVDF membranes at 200 mA for 2 h. The primary antibodies used in this study were: α-PHIP (1:1000, Bethyl laboratories, A302-055A), α-PHIP-N (1:1000), α-SP3 (1:1000, Santa Cruz, sc-655), α-actin (1:20000, Sigma, A5316), or α-eEF2 (1:1000, Cell Signaling, 2332). Secondary α–rabbit and α-mouse antibodies (Jackson Immunoresearch, 1:5000, 715-035-146/152) were used and the signal was visualized using enhanced chemiluminescence (ECL) (Perkin Elmer).

## Additional Information

**How to cite this article**: Katigbak, A. *et al*. A CRISPR/Cas9 Functional Screen Identifies Rare Tumor Suppressors. *Sci. Rep.* **6**, 38968; doi: 10.1038/srep38968 (2016).

**Publisher's note:** Springer Nature remains neutral with regard to jurisdictional claims in published maps and institutional affiliations.

## Supplementary Material

Supplementary Figures

Supplementary Table S1
