# Supplementary material for: A CRISPR/Cas9 Functional Screen Identifies Rare Tumor Suppressors
Source: Sci Rep. 2016 Dec 16;6:38968. doi: 10.1038/srep38968 (PMC5159885; doi:10.1038/srep38968)
Supplement: Supplementary Figures [file srep38968-s1.pdf]

A CRISPR/Cas9 Functional Screen Identifies Rare Tumor Suppressors

Alexandra Katigbak, Regina Cencic, Francis Robert, Patrick Sénéchal, Claudio Scuoppo, Jerry Pelletier

SUPPLEMENTARY FIGURES AND TABLES

SUPPLEMENTARY FIGURES

Supplementary Figure 1. a. Flow chart denoting steps used to identify the gene set targeted in this study. **b.** T7EI assay performed on DNA isolated from NIH 3T3 cells infected with pQCiG2 derivatives harboring sgRNAs to the indicated loci. NT, non-transduced.

Supplementary Figure 2. a. Kaplan-Meier plot of tumor onset rates in mice transplanted with HSPCs transduced with pQCiG2/sgp53-1, pQCiG2/sgRosa26, and the indicated dilutions of pQCiG2/sgp53-1 with pQCiG2/sgRosa26. **b.** Kaplan-Meier plot of tumor onset rates in mice transplanted with HSPCs infected with the indicated sgRNA pools. Note that data from all cohorts receiving the sgRosa26 pool is combined and used as reference in these plots. In parenthesis are the p values (relative to sgRosa26 cohort) as determined by the Log-Rank Mantel-Cox Test.

Supplementary Figure 3. Sequence analysis of the *Sp3* locus from sgSp3-derived tumors. PCR products obtained following amplification of the *Sp3* locus were cloned into pSKII(+) and sequenced. The region targeted by the sgRNA is highlight in light blue and the PAM motif is denoted in red. Dashes represent deleted nucleotides and the number of clones harboring the indicated lesions is denoted to the right. The presence of wild-type alleles likely reflects contamination of the tumor by infiltrating wild-type cells or hemizygous inactivation being sufficient to drive tumor initiation.

Supplementary Figure 4. Sequence analysis of the *Phip* locus from sgPhip-derived tumors. PCR products obtained following amplification of the *Phip* locus were cloned into pSKII(+) and sequenced. The region targeted by the sgRNA is highlighted in light blue and the PAM motif is denoted in red. Dashes represent deleted nucleotides and the number of clones harboring the indicated lesions is denoted to the right. The presence of wild-type alleles may reflect contamination of the tumor by infiltrating normal cells or hemizygous inactivation being sufficient to cooperate with MYC over-expression in lymphomagenesis.

Supplementary Figure 5. Functional assessment of a PHIP truncation mutant in the Eμ-Myc model. **a.** Schematic diagram showing functional domains of PHIP and the site of the PHIP$^{R1212\Delta}$ truncation mutation. **b.** Immunoblot illustrating ectopic expression of full-length PHIP and PHIP$^{R1212\Delta}$ in NIH 3T3 cells following retroviral transduction. Solid and white arrowheads indicate the position of migration of PHIP and PHIP$^{R1212\Delta}$, respectively. **c.** Kaplan-Meier plot of tumor onset in mice receiving HSPCs transduced with retrovirus expressing PHIP or PHIP$^{R1212\Delta}$.

Supplementary Figure 6. Frequency of mutations in *PHIP* (**a**) and *SP3* (**b**) across human tumor samples as reported from the COSMIC (v77) database (http://cancer.sanger.ac.uk/cosmic).

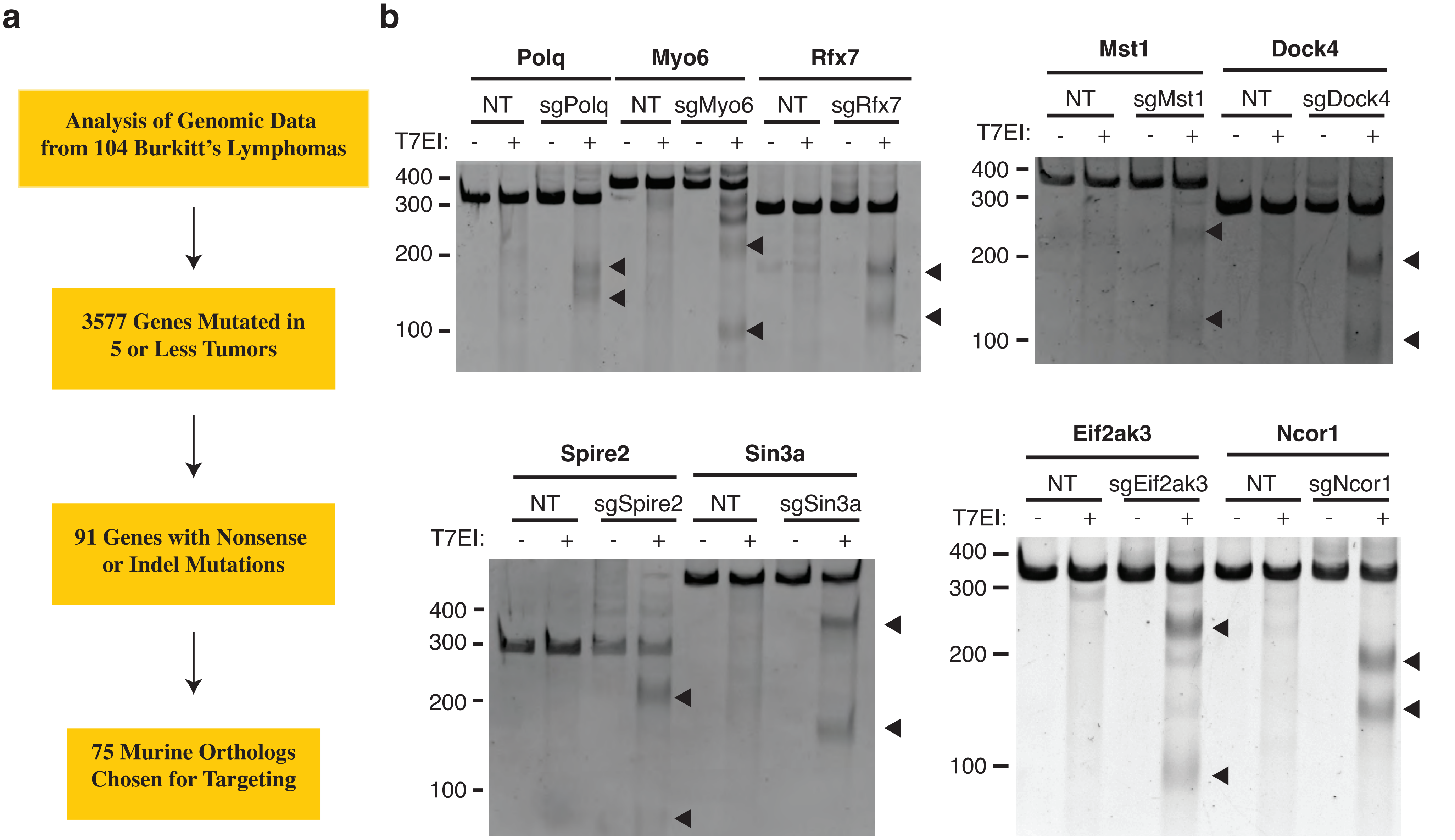

Supplementary Figure 1

a

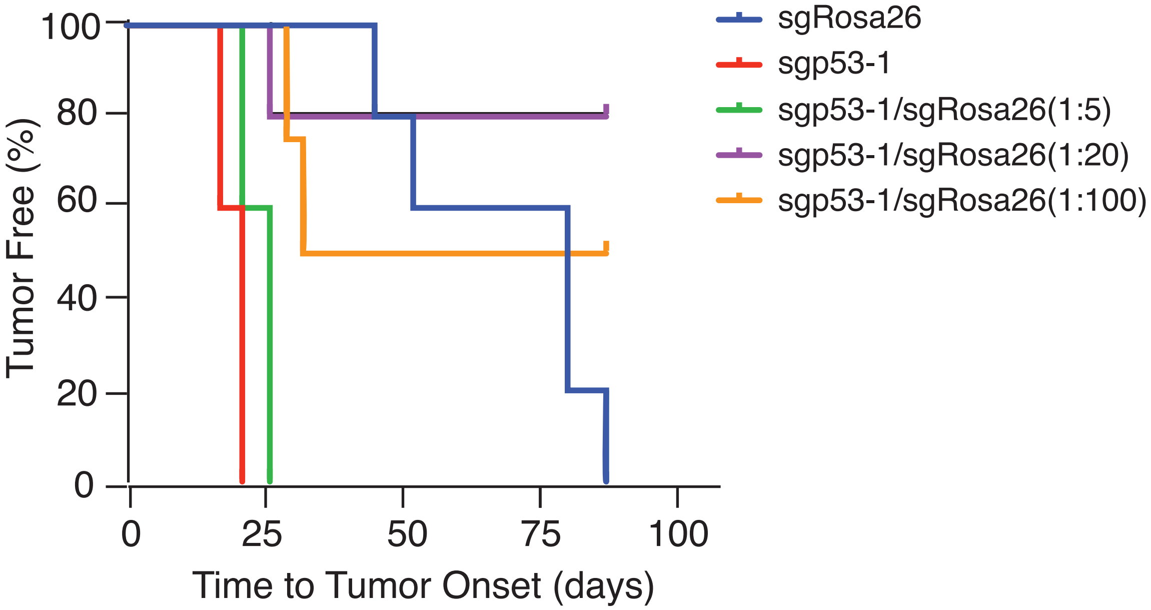

b

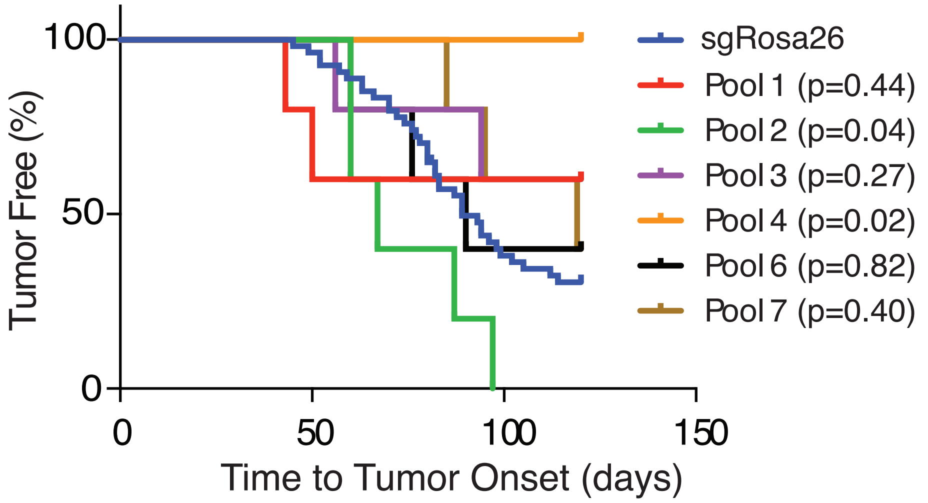

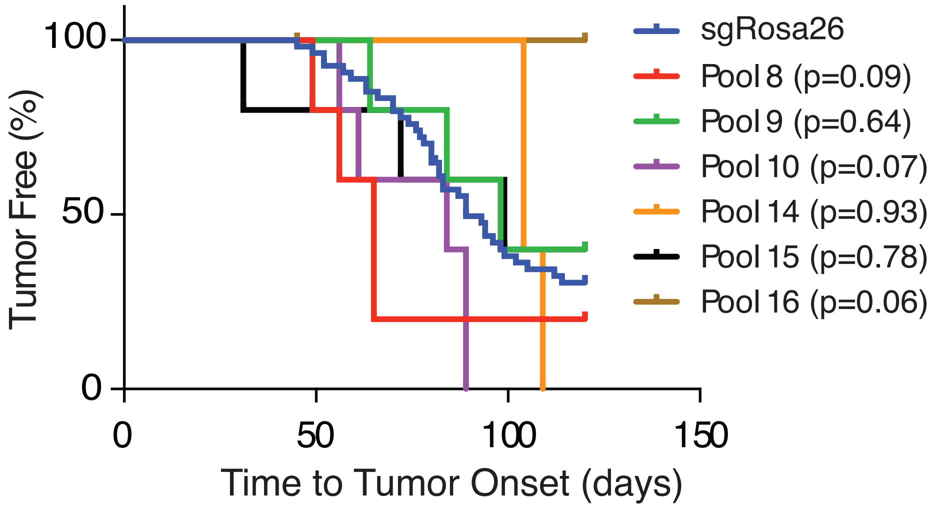

Supplementary Figure 2

```
Sp3 Locus                Guide Sequence      PAM                                  # of Clones

WT   CTCACATCTGAGAGCACACCTGCGTTGGCA--TTCGGGGGAGCGCCCTTTTATTTGTAAC   7
T1   CTCACATCTGAGAGCACACCTGCGTTGGCAT-TTCGGGGGAGCGCCCTTTTATTTGTAAC   16
     CTCACATCTGAGAGCACACCTGCGTTGGCAG-TTCGGGGGAGCGCCCTTTTATTTGTAAC   14
     CTCACATCTGAGAGCACACCTGCGTTGGCACCTTCGGGGGAGCGCCCTTTTATTTGTAAC   12
     CTCACATCTGAGAGCACACCTGCGTTGGCAA-TTCGGGGGAGCGCCCTTTTATTTGTAAC   3
     CTCACATCTGAGAGCAC----------------------------CCTTTTATTTGTAAC   2
     CTCACATCTGAGAGCACACCTGCGTTGGCAC-TTCGGGGGAGCGCCCTTTTATTTGTAAC   1

WT   CTCACATCTGAGAGCACACCTGCGTTGGCA----TTCGGGGGAGCGCCCTTTTATTTGTAAC   3
T2   CTCACATCTGAGAGCACACCTGCGTTGGCAA---TTCGGGGGAGCGCCCTTTTATTTGTAAC   50
     CTCACATCTGAGAGCACACCTGCGTTGGCAAC--TTCGGGGGAGCGCCCTTTTATTTGTAAC   8
     CTCACATCTGAGAGCAC------------------------------CCTTTTATTTGTAAC   2
     CTCACATCTGAGAGCACACCTGCGTTGGCAAT--TTCGGGGGAGCGCCCTTTTATTTGTAAC   1
     CTCACATCTGAGAGCACACCTGCGTTGGCAGAT-TTCGGGGGAGCGCCCTTTTATTTGTAAC   1
     CTCACATCTGAGAGCACACCTGCGTTGGCAGGTTTTCGGGGGAGCGCCCTTTTATTTGTAAC   1

WT   CTCACATCTGAGAGCACACCTGCGTTGGCA----TTCGGGGGAGCGCCCTTTTATTTGTAAC   3
T3   CTCACATCTGAGAGCACACCTGCGTTGGCAA---TTCGGGGGAGCGCCCTTTTATTTGTAAC   45
     CTCACATCTGAGAGCACACCTGCGTTGGCAGGACTTCGGGGGAGCGCCCTTTTATTTGTAAC   20
     CTCACATCTGAGAGCACACCTGCGTTGGCAG---TTCGGGGGAGCGCCCTTTTATTTGTAAC   2

WT   TAAAGTCTATGGGAAGACCTCACATCTGAGAGCACACCTGCGTTGGCA-----TTCGGGGGAGCGCCCTTTTATTTGTAACTGG   8
T4   TAAAGTCTATGGGAAGACCTCACATCTGAGAGCACACCTGCGTTGGCAA----TTCGGGGGAGCGCCCTTTTATTTGTAACTGG   19
     TAAAGTCTATGGGAAGACCTCACATCTGAGAGCACACCTGCGTTGGCAGT---TTCGGGGGAGCGCCCTTTTATTTGTAACTGG   13
     TAAAGTCTATGGGAAGACCTCACATCTGAGAGCACACCTGCGTTGGCAGGGCCTTCGGGGGAGCGCCCTTTTATTTGTAACTGG   11
     TAAAGTCTATGGGAAGACCTCACATCTGAGAGCACACCTGCGTTGGCAGG---TTCGGGGGAGCGCCCTTTTATTTGTAACTGG   9
     TAA-----------------------------------------------------------------------------CTGG   8
     TAAAGTCTATGGGAAGACCTCACATCTGAGAGCACACCTGCGTT------------GGGGGAGCGCCCTTTTATTTGTAACTGG   4
     TAAAGTCTATGGGAAGACCTCACATCTGAGAGCACACCTGCGTTGGCAT----TTCGGGGGAGCGCCCTTTTATTTGTAACTGG   2
     TAAAGTCTATGGGAAGACCTCACATCTGAGAGCACACCTGCGTTGGCAC----TTCGGGGGAGCGCCCTTTTATTTGTAACTGG   1
     TAAAGTCTATGGGAAGACCTCACATCTGAGAGCACACCTGCGTTGGCACC---TTCGGGGGAGCGCCCTTTTATTTGTAACTGG   1
     TAAAGTCTATGGGAAGACCTCACATCTGAGAGC---------------------------------CCTTTTATTTGTAACTGG   1

WT   CCTCACATCTGAGAGCACACCTGCGTTGGCATTCGGGGGAGCGCCCTTTTATTTGTAACT   2
T5   CCTCACATCTGAGAGCACACCTGCGTT------CGGGGGAGCGCCCTTTTATTTGTAACT   35
     CCTCACATCTGAGAGCACACCTGCGTTGGCA-GAGGGAGAGCGCCCTTTTATTTGTAACT   31
```

Supplementary Figure 3

Phip Locus	Guide Sequence / PAM	# of Clones
WT	CCTTATTTATTTTAGATATTGCGTCTGCATTTGTTGCCCCTGTGGACCTTCAAGCTTATCCCATGTATTGCACTGTGG	28
T1	CCTTATTTATTTTAGATATTGCGTCTGCATTTGTTGCCC-TGTGGACCTTCAAGCTTATCCCATGTATTGCACTGTGG	2
	CCTTATTTATTTTAGATATTGCGTCTGCATTTGTTGCC--TGTGGACCTTCAAGCTTATCCCATGTATTGCACTGTGG	1
	CCTTATTTATTTTAGATATTGCGTCTGCATTTG---------TGGACCTTCAAGCTTATCCCATGTATTGCACTGTGG	1
	CCTTATTTATTTTAGATATTGCGTCTGCATT---------TGTGGACCTTCAAGCTTATCCCATGTATTGCACTGTGG	1
	CCTTATTTATTTTAGATATTGCGTCTGCATT--------------------AAGCTTATCCCATGTATTGCACTGTGG	1
	CCTTATTTATTTTAGATATTGCGTCT-----------------------TCAAGCTTATCCCATGTATTGCACTGTGG	1
	CCTTATTTATTTTAGATAT------------------------------TCAAGCTTATCCCATGTATTGCACTGTGG	1
	CCTTATTTATTTTAGATATTGCGTCTGCA--ATTATCTTAATAT--------AGCTTATCCCATGTATTGCACTGTGG	1
	CCTTATTTATTTTAGATATT---GCACTGTGG	1
	CCTT-TT--GCACTGTGG	1
T2	CCTTATTTATTTTAGATATTGCGTCTGCATTTGTTGCCCCTGTGGACCTTCAAGCTTATCCCATGTATTGCACTGTGG	12
	CCTTATTTATTTTAGATATTGCGTCTGCATTTGTTGCCC-TGTGGACCTTCAAGCTTATCCCATGTATTGCACTGTGG	26
	CCTTATTTATTTTAGATATTGCGTCTGCATTTGTTGCCC--GTGGACCTTCAAGCTTATCCCATGTATTGCACTGTGG	12
	CCTTATTTATTTTAGATATTGCGTCTGCATTTG-----------GACCTTCAAGCTTATCCCATGTATTGCACTGTGG	8
	CCTTATTTATTTTAGATATTGCGTCTGCATT-------CCTGTGGACCTTCAAGCTTATCCCATGTATTGCACTGTGG	3
	CCTTATTTATTTTAGATATTGCGTCTGCATT-------CCTGTGGACCTTCAAGCTTATCCCATGTATTGCACTGTGG	3
	CCTTATTTATTTTAGATATTGCGTCTGCATTTGT---------GGACCTTCAAGCTTATCCCATGTATTGCACTGTGG	2
	CCTTATTTATTTTAGATATTGCGTCTGCATTTGTTGCCC--ATGGACCTTCAAGCTTATCCCATGTATTGCACTGTGG	1
	CCTTATTTATTTTAGATATTGCGTCTGCATT-------------GACCTTCAAGCTTATCCCATGTATTGCACTGTGG	1
T3	CCTTATTTATTTTAGATATTGCGTCTGCATTTGTTGCCCCTGTGGACCTTCAAGCTTATCCCATGTATTGCACTGTGG	2
	CCTTATTTATTTTAGATATTGCGTCTGCATTTGTTGCCC-TGTGGACCTTCAAGCTTATCCCATGTATTGCACTGTGG	10
	CCTTATTTTATTTTAGATATTGCGTCTGCATTTG----------GGACCTTCAAGCTTATCCCATGTATTGCACTGTGG	10
T4	CCTTATTTATTTTAGATATTGCGTCTGCATTTGTTGCCCCTGTGGACCTTCAAGCTTATCCCATGTATTGCACTGTGG	1
	CCTTATTTATTTTAGATATTGCGTCTGCATTTGTTGCCC-TGTGGACCTTCAAGCTTATCCCATGTATTGCACTGTGG	10
	CCTTATTTATTTTAGATATTGCGTCTGCATTTGT-----CTGTGGACCTTCAAGCTTATCCCATGTATTGCACTGTGG	4
	CCTTATTTATTTTAGATAT---------ATAAG--------GTGGACCTTCAAGCTTATCCCATGTATTGCACTGTGG	3
T5	CCTTATTTATTTTAGATATTGCGTCTGCATTTGTTGCCCCTGTGGACCTTCAAGCTTATCCCATGTATTGCACTGTGG	3
	CCTTATTTATTTTAGATATTGCGTCTGCATTTGTTGCCCCCTGTGGACCTTCAAGCTTATCCCATGTATTGCACTGTGG	17
	CCTTATTTATTTTAGATATTGCGTCTGCATTTGTTGCCC-TGTGGACCTTCAAGCTTATCCCATGTATTGCACTGTGG	4
	CCTTATTTATTTTAGATATTGCGTCTGCATTATCTTATCCCAT----------GCTTATCCCATGTATTGCACTGTGG	1
T6	CCTTATTTATTTTAGATATTGCGTCTGCATTTGTTGCCCCTGTGGACCTTCAAGCTTATCCCATGTATTGCACTGTGG	3
	CCTTATTTATTTTAGATATTGCGTCTGCATTTGTTGCCCCCTGTGGACCTTCAAGCTTATCCCATGTATTGCACTGTGG	25
	CCTTATTTATTTTAGATATTGCGTCTGCATTTGTTGCCC-TGTGGACCTTCAAGCTTATCCCATGTATTGCACTGTGG	5
	CCTTATTTATTTTAGATATTGCGTCTGCATTTGT-----CTGTGGACCTTCAAGCTTATCCCATGTATTGCACTGTGG	1
	CCTTATTTATTTTAGATATTG--TATTGCACTGTGG	1

Supplementary Figure 4

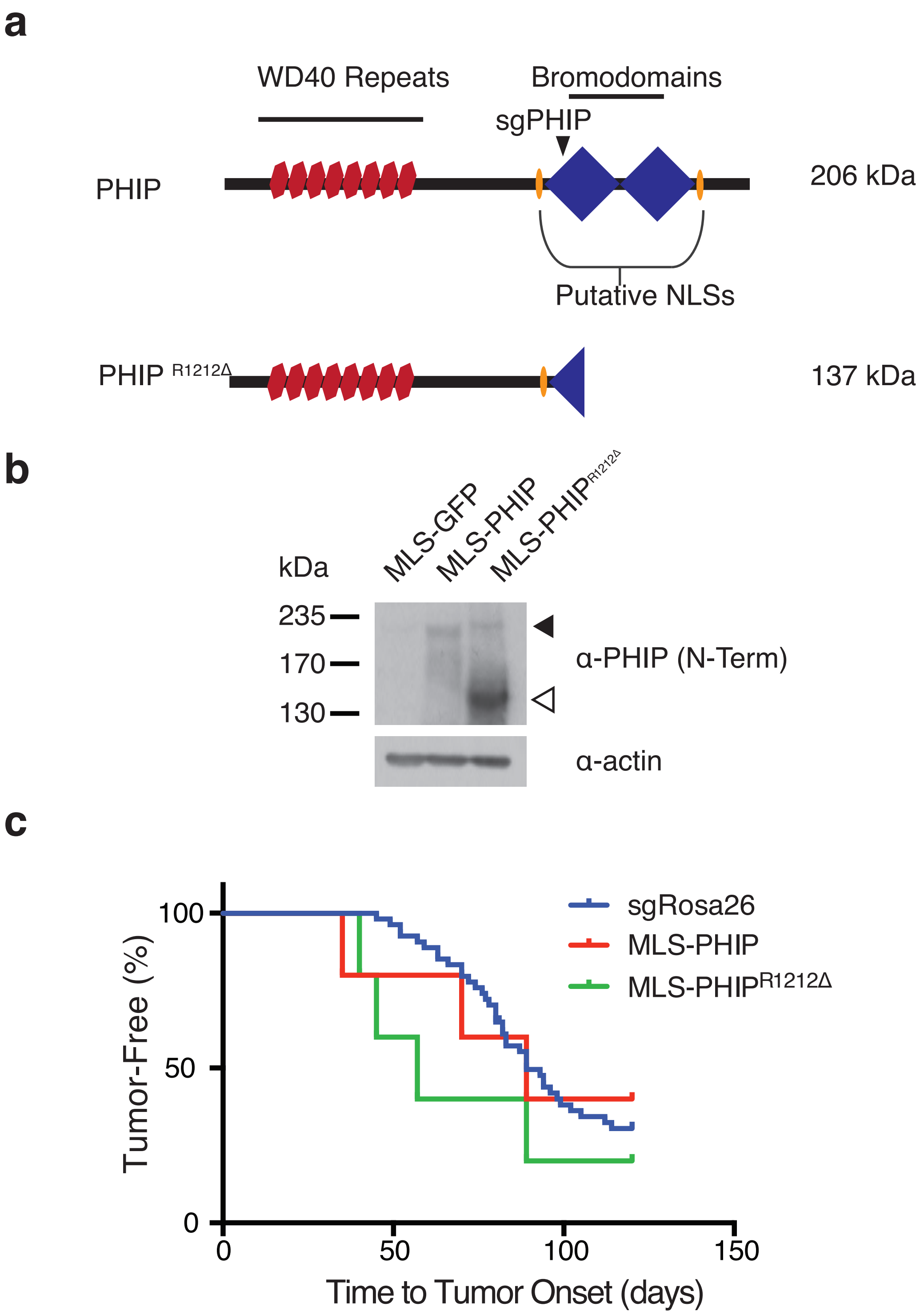

Supplementary Figure 5

a

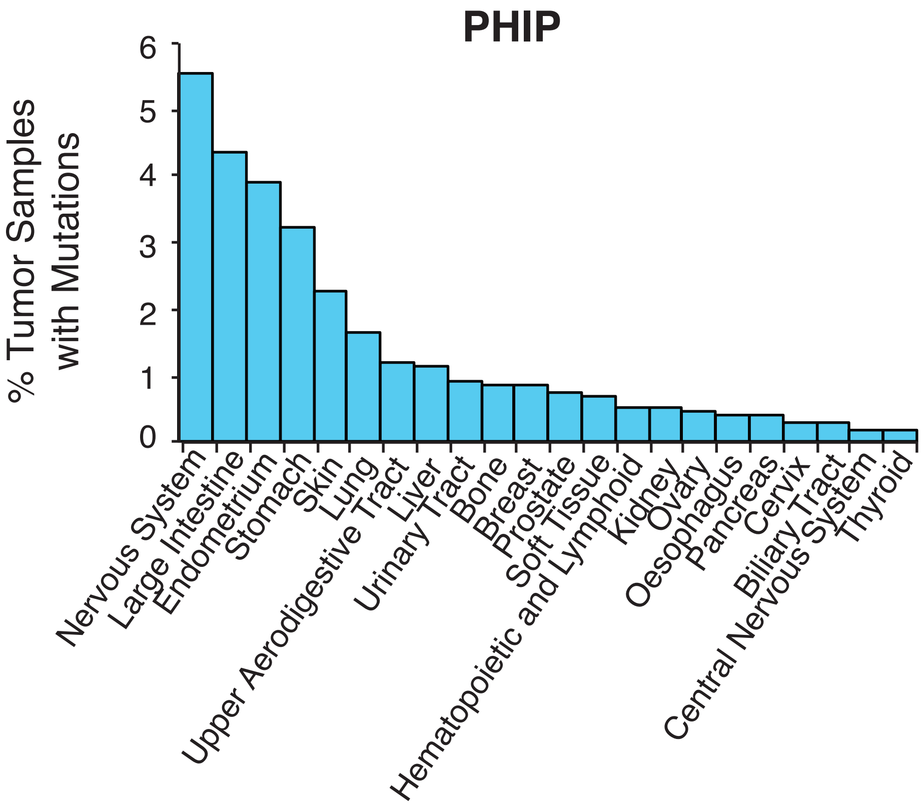

b

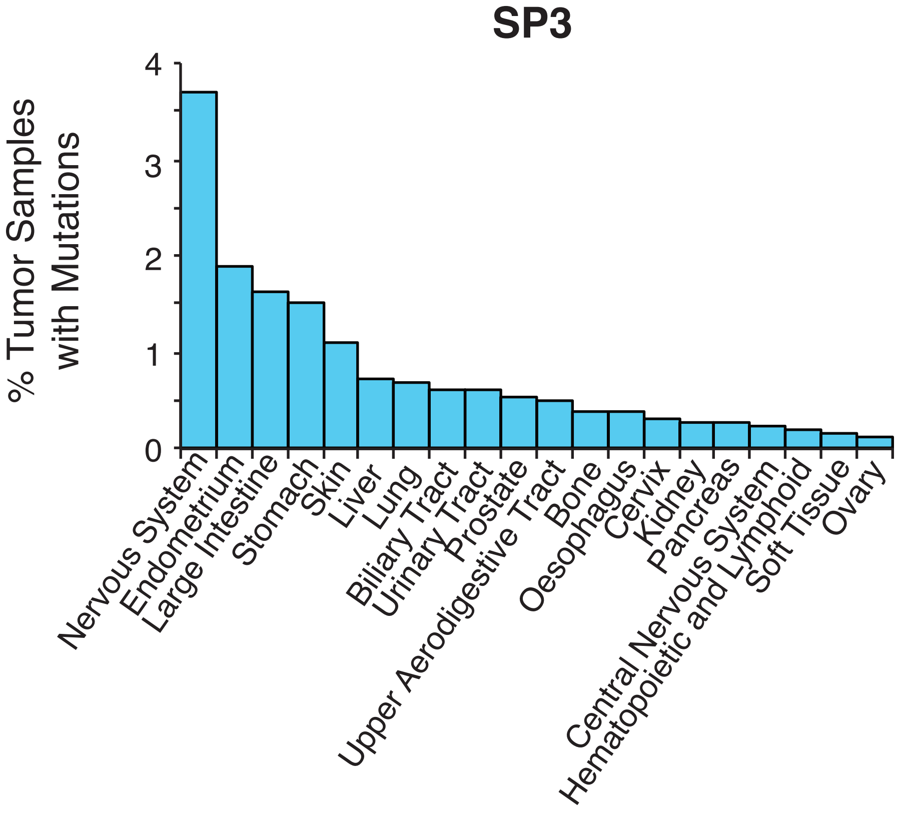

Supplementary Figure 6

Supplemental Table 2. List of sgRNA and shRNAs used to validate candidate tumor suppressor genes.

**sgRNA Name**	**sgRNA Sequence**	
sgPHIP-2	GTATCCCATGTATTGCACTG	
sgSP3-2	TTTGTAACTGGATGTTCTG	
sgTFAP4-2	GAGAAAGAAGTGATAGGA	
**shRNA Name**	**shRNA Oligonucleotide**	**shRNA Target Site**
shPHIP-3809	TGCTGTTGACAGTGAGCGCACGGATCTAAGTACAATTAAATAGTGAAGCCACAGATGTATTTAATTGTACTTAGATCCGTTTGCCTACTGCCTCGGA	CGGATCTAAGTACAATTAAATA
shPHIP-4131	TGCTGTTGACAGTGAGCGCACAGAGCTCAGTCTTACGATATAGTGAAGCCACAGATGTATATCGTAAGACTGAGCTCTGTTTGCCTACTGCCTCGGA	CAGAGCTCAGTCTTACGATATA
shSP3-658	TGCTGTTGACAGTGAGCGCCCAATCAATAGTGTCGATCTATAGTGAAGCCACAGATGTATAGATCGACACTATTGATTGGTTGCCTACTGCCTCGGA	CAATCAATAGTGTCGATCTATA
shSP3-3117	TGCTGTTGACAGTGAGCGCTCGTTGTAAATTACCAATAAATAGTGAAGCCACAGATGTATTTATTGGTAATTTACAACGATTGCCTACTGCCTCGGA	CGTTGTAAATTACCAATAAATA

Supplemental Table 3. PCR Primer sequences used in this study

ID	Sequence
sgRNA-ID-F	AGCCCTTTGTACACCCTAAGCCTC
sgRNA-ID-R	CTAACTGACACACATTCCACAGGG
PHIP-F	CCATCTCATCCCTGCGTGTCTCCGACTCAGTGTAATTTCTTCATCCTAATGTACCA
PHIP-R	CCTCTCTATGGGCAGTCGGTGATGTTGGATAGGCCACCACAGT
SP3-F	CCATCTCATCCCTGCGTGTCTCCGACTCAGTGGGAAAAAGAAGCAACACA
SP3-R	CCTCTCTATGGGCAGTCGGTGATGCCTCTGTAATTCATCACTTCG
TSC1-F	CCATCTCATCCCTGCGTGTCTCCGACTCAGTGCTTGTCAACACGTTGGTT
TSC2-1	CCTCTCTATGGGCAGTCGGTGATCTATGGATGAGCTGCTGTGG
Sin3a-F	TCAGCTGTGCCACAAAGTTC
Sin3a-R	TGTGCCCAGACATGTGTACT
Myo6-F	AGTCCACCATGATGACGAGG
Myo6-R	CTGGGCTCCACTCTGAAACT
Dock4-F	GTTTCTCTTCCCAGCTTCGC
Dock4-R	AGGATGAGTCAGATGGTGCT
Mst1-F	AGCACTGGTTTTGGCTCAAG
Mst1-R	TGGGTATAGCAGGCAAGTGG
Polq-F	TGGTTCTGTGGTAATGATTTTGG
Polq-R	AGCTCTTACTGGTCAACTTTCA
Spire2-F	TCAGAAGTGGCAGGACAAGG
Spire2-R	TTGAGAGTCCTGGTGTTGGG
Eif2ak3-F	CCTCGTGACGCTTGTTTTCT
Eif2ak3-R	TCTGGTAAGTCTGAGTGCCG
Rfx7-F	GTGAACCCTGCTCTTGTCAC
Rfx7-R	TGGCTGTATGTGTCCTGTGG
Ncor1-F	ACCCAGAAATGCAGGTACCA
Ncor1-R	ACCAAAGCCACACAATTGCT
